# Conversion of Human Fibroblasts into Induced Neural Stem Cells by Small Molecules

**DOI:** 10.3390/ijms23031740

**Published:** 2022-02-03

**Authors:** Donghui Liu, Grigori Rychkov, Plinio Hurtado, Hai-Yun Luo, Tao Zhang, Larisa Bobrovskaya, Xin-Fu Zhou

**Affiliations:** 1 Health and Biomedical Innovation, Clinical and Health Sciences, University of South Australia, Adelaide, SA 5000, Australia; liudy016@mymail.unisa.edu.au (D.L.); grigori.rychkov@adelaide.edu.au (G.R.); larisa.bobrovskaya@unisa.edu.au (L.B.); 2 School of Biomedicine, University of Adelaide, Adelaide, SA 5000, Australia; 3 South Australian Health and Medical Research Institute (SAHMRI), Adelaide, SA 5000, Australia; 4 Department of Renal Medicine, The Royal Adelaide Hospital, Adelaide, SA 5000, Australia; plinio.hurtado@adelaide.edu.au; 5 Adelaide Medical School, University of Adelaide, Adelaide, SA 5000, Australia; 6 Department of Pharmacology, College of Basic Medicine, Kunming Medical University, Kunming 650500, China; luohaiyun12@163.com (H.-Y.L.); zhangang0118@163.com (T.Z.)

**Keywords:** small molecules, induced neuron stem cells, human fibroblasts

## Abstract

Induced neural stem cells (iNSCs) reprogrammed from somatic cells hold great potentials for drug discovery, disease modelling and the treatment of neurological diseases. Although studies have shown that human somatic cells can be converted into iNSCs by introducing transcription factors, these iNSCs are unlikely to be used for clinical application due to the safety concern of using exogenous genes and viral transduction vectors. Here, we report the successful conversion of human fibroblasts into iNSCs using a cocktail of small molecules. Furthermore, our results demonstrate that these human iNSCs (hiNSCs) have similar gene expression profiles to bona fide NSCs, can proliferate, and are capable of differentiating into glial cells and functional neurons. This study collectively describes a novel approach based on small molecules to produce hiNSCs from human fibroblasts, which may be useful for both research and therapeutic purposes.

## 1. Introduction

Neurodegeneration diseases, such as Alzheimer’s Disease (AD), Parkinson’s Disease (PD) and stroke, remain major health problems given the limited therapeutic options available. Although many conventional therapies can alleviate the disease symptoms, most of the treatments fail to reverse or slow the progression of these diseases. However, recent developments in cell-based therapies have brought new hopes for treating neurological disorders, especially for neurodegenerative diseases. Studies have shown that neural cell transplantation can effectively treat neurodegeneration [1,2,3]. However, the availability of transplantable neural cells in terms of cell source and cell quantity remains a major hurdle to overcome. It has been proven that cell fate is reversible since researchers have successfully converted somatic cells into induced pluripotent stem cells (iPSCs) [4,5]. The established iPSCs technology provides an opportunity to generate iPSCs from autologous somatic cells suitable for patient-specific therapy. However, clinical use of iPSCs is challenging due to the time-consuming induction, indeterminate differentiation, and potential tumorigenicity of the cells [6,7]. Recently, several studies have reported that somatic cells can be directly reprogrammed into induced neural stem cells (iNSCs) bypassing pluripotent stage [8]. Compared to iPSCs, iNSCs are easier to terminally differentiate into neurons, astrocytes, and oligodendrocytes, and, therefore, may be better suited for the treatment of neurological diseases. However, many of the studies are based on the introduction of exogenous transcription factors which pose safety concerns of exogenous random integration [9,10,11]. Therefore, it has become a hotspot for scientists to develop non-integrative methods for cell reprogramming, such as using recombinant proteins [12], miRNAs [13], and small molecules [14,15,16]. Of all current methods, small molecules are preferred due to their reported safety and effectiveness in cell reprogramming, and the fact that they can be easily introduced and manipulated.

In this study, we attempted to generate neural stem cells from human fibroblasts using a cocktail of small molecules in combination with basic medium and pro-neural factors.

## 2. Results

### 2.1. Generation of hiNSCs from Human Fibroblasts by Small Molecules

**Figure 1:**
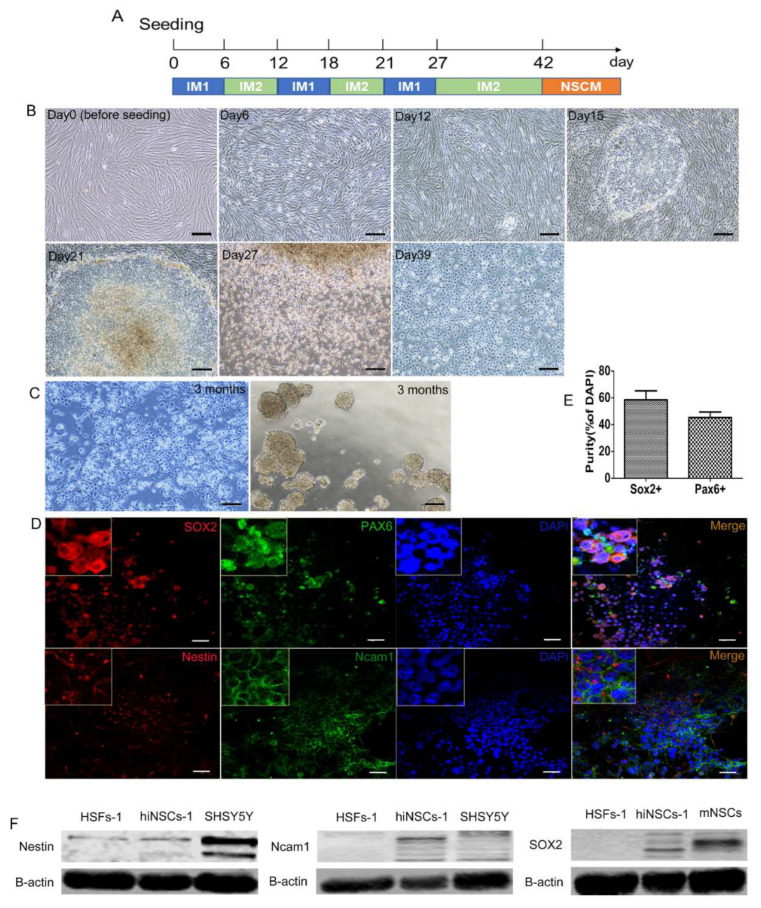
Generation of human iNSCs from fibroblasts by small molecules. (**A**) Scheme of induction procedure. See Methods and Supplemental Experimental Procedures for detailed protocol. (**B**) Phase contrast images of cells during induction time, scale bars are 200 μm. (**C**) Morphology of hiNSCs-1 in monolayer culture and suspension culture, scale bars are 200 μm. (**D**) hiNSCs-1 stained for neural stem cell makers, SOX2, Nestin, PAX6, and Ncam1, scale bars are 40 μm. (**E**) The percentage of Sox2+ and Pax6+ cells in total cells at the time of quantification (means ± SEM, cell counting was from 3 triplicate samples). (**F**) Representative immunoblots for SOX2, Nestin (upper band), and Ncam1 in different cells, B-actin (beta-actin) was used as a loading control.

We have previously reported that mouse fibroblasts could be reprogrammed into iNSCs by a cocktail of small molecules containing VPA, Bix01294, RG108, PD0325901, CHIR99021, Ascorbic acid, and A83-01, or another cocktail containing Bix01294, RG108, and PD0325901 [16]. However, our attempts to use these protocols to induce iNSCs from human fibroblasts were unsuccessful. In a recent review, we listed various small molecules that have been reported to facilitate neural stem cell induction [17]. Based on this analysis, here, we tested different combinations of small molecules for the induction of iNSCs from human skin fibroblasts (HSFs-1). We found that a combination of Bix01294, RG108, CHIR99021, Ascorbic acid, Repsox, LDN193189, and Y27632 stimulated generation of a distinct cell colony from HSFs in 12 days (Figure 1A,B). To reduce cell apoptosis a pan-caspase inhibitor, Q-VD-OPh, was added to the growing medium. We found these induced cells were able to proliferate under the induction procedure, while most of HSFs-1 were dead at Day 27 (see Section 4 and Supplemental Experimental Procedures for detailed protocol). After passaging, these induced cells became morphologically stem cell-like and homogeneous, and almost no fibroblast-like morphology has been observed. After 42 days, the induced cells were cultured in neural stem cell medium (NSCM) for months without small molecules. The stem cell-like morphology was stable under monolayer culture, and cells were able to form spheres under suspension culture, which is a salient property of neural stem cells (NSCs) (Figure 1C). To examine if these induced cells expressed NSC specific genes, several common NSC makers (SOX2, Nestin, PAX6, and Ncam1) were selected for immunocytochemistry staining. The results showed that these cells were positive for SOX2, Nestin, PAX6, and Ncam1 (Figure 1D,E). Moreover, the results from western blots also confirmed the upregulated expression of SOX2, Nestin, and Ncam1 of the induced cells (termed hiNSCs-1 hereafter), compared to HSFs-1 (Figure 1F). Together, these data indicate that a combination of Bix01294, RG108, CHIR99021, Ascorbic acid, Repsox, LDN193189, Y27632, and Q-VD-OPh can generate hiNSCs which express key NSC makers from HSFs.

### 2.2. hiNSCs-1 Express Multiple Up-Regulated NSCs Makers and Down-Regulated Fibroblast-Specific Genes

**Figure 2:**
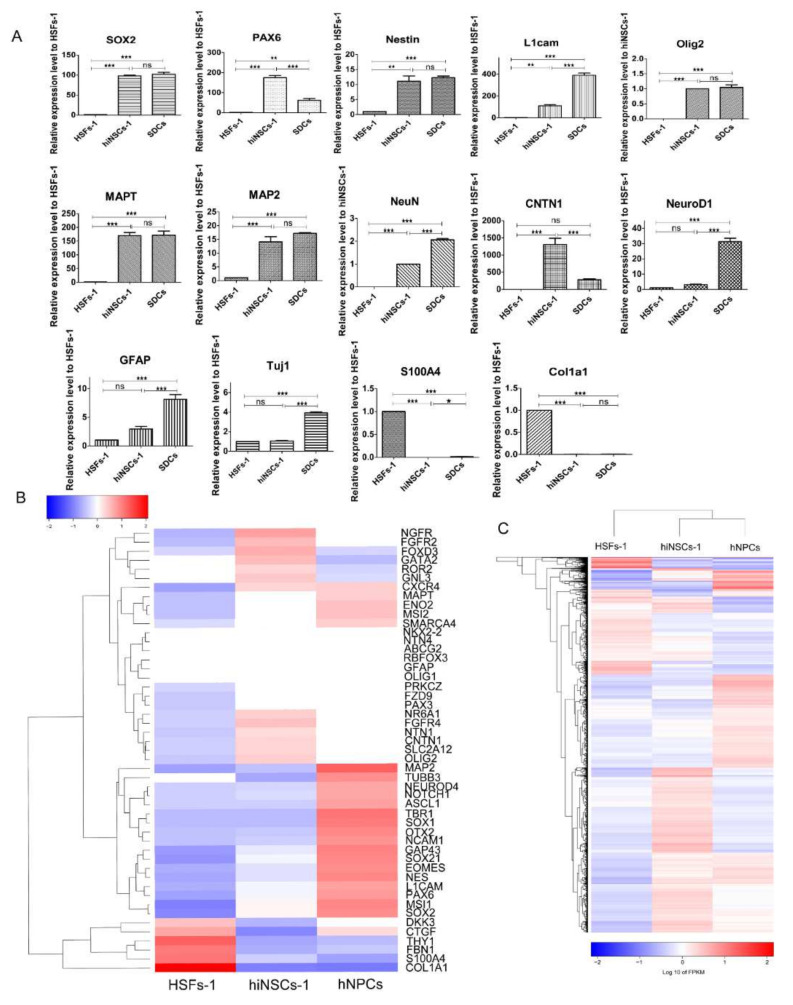
hiNSCs-1 express multiple up-regulated NSCs makers and down-regulated fibroblast-specific genes. (**A**) qRT-PCR results of the expression of some neural-lineage makers and fibroblast-specific genes in HSFs-1, hiNSCs-1 and spontaneous differentiated cells (SDCs). * *p* < 0.05, ** *p* < 0.01, *** *p* < 0.001, ns = not significant. (**B**) Heatmap of the expression of neural lineage makers and fibroblast-specific genes in HSFs-1, hiNSCs-1, and hNPCs using RNA-sequencing. (**C**) Heatmap and hierarchical clustering of genes with significance in HSFs-1, hiNSCs-1, and hNPCs using RNA-sequencing.

To further characterize the phenotype of the hiNSCs-1, we quantitatively analyzed the expression of several neural-lineage makers and fibroblast-specific genes using Reverse Transcription Polymerase Chain Reaction (RT-PCR) (Figure 2A). The results showed that the expression of neural-lineage makers (SOX2, PAX6, Nestin, Olig2, L1cam, MAPT, CNTN1, MAP2, and NeuN) was significantly upregulated in hiNSCs-1 compared to HSFs-1, while the expression of fibroblast-specific genes (Col1a1 and S100A4) was significantly down-regulated. We then examined the quantity and sequences of RNA in the fibroblasts and hiNSCs using RNA-sequencing technique. The heatmap analysis showed that hiNSCs-1 expressed multiple neural lineage makers, but not fibroblast-specific genes (DKK, CTGF, THY1, FBN1, S100A4, and COL1A1), which is consistent with the RT-PCR results (Figure 2B). Moreover, the heatmap and hierarchical clustering of genes with significance showed that the gene expression pattern of hiNSCs-1 was more similar to that of human neural progenitor cells (hNPCs, datasets from NCBI, GEO accession: GSM2073121), rather than that of human fibroblasts (Figure 2C). However, it is noteworthy that there are still big differences of gene expression between hiNSCs and hNPCs as the heatmaps have shown, this may be due to the incomplete reprogramming of hiNSCs-1 or/and the different developmental stage between hiNSCs and hNPCs. Overall, these data indicate that hiNSCs-1 express multiple up-regulated NSCs makers and down-regulated fibroblast-specific genes.

### 2.3. hiNSCs-1 Can Differentiate into Astrocytes, Oligodendrocytes, and Neurons In Vitro

**Figure 3:**
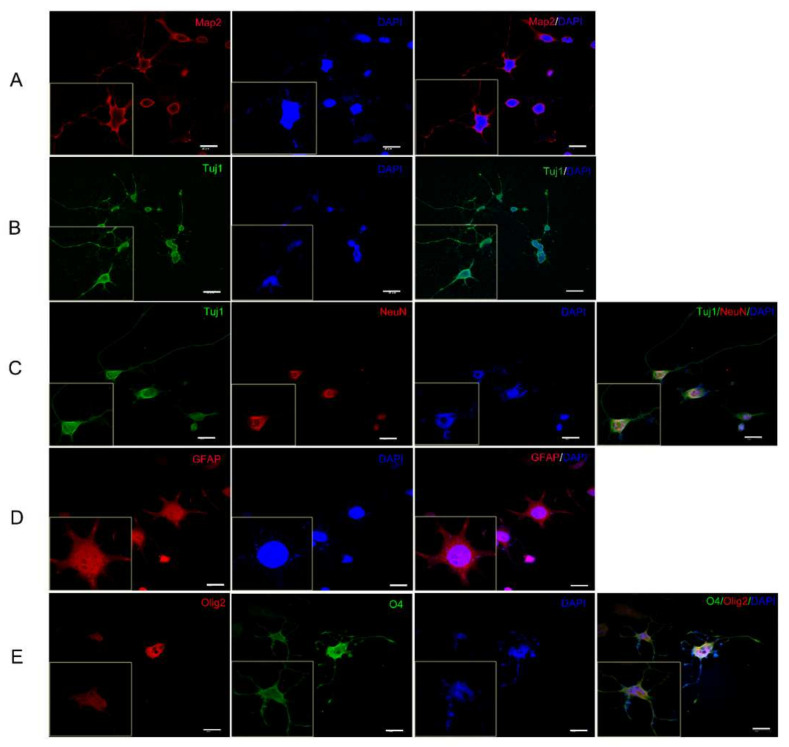
Differentiation of hiNSCs-1 in vitro. hiNSCs-1 differentiated into neurons (marked by Tuj1, Map2, and NeuN, **A**–**C**), astrocytes (marked by GFAP, **D**), and Oligodendrocytes (marked by O4 and Olig2, **E**) after 2–3 weeks culture in NDM, ADM, and ODM, respectively. Scale bars are 40 μm.

hiNSCs-1 showed a similar gene expression pattern with hNPCs; therefore, we asked if these cells could differentiate into astrocytes, oligodendrocytes, and neurons, as hNPCs would. It was found, however, that these cells had strong proliferation ability that impeded their differentiation (data not shown). Therefore, a DNA alkylating and crosslinking agent mitomycin C (MMC) has been used to inhibit their proliferation. After treating with MMC (10 ug/mL) for 1 h, hiNSCs-1 were cultured in neural differentiation medium for 10 days to 6 weeks according to each analysis. To examine the spontaneous differentiation of the cells, hiNSCs-1 were cultured in neural stem cell medium (NSCM) without EGF and bFGF for 10 days, and the expression of several neural markers was quantified using RT-PCR (Figure 2A). The results showed that the expression of neuronal markers NeuN, NeuroD1 and Tuj1, and astrocyte marker GFAP were significantly upregulated in spontaneous differentiated cells (SDCs) compared to hiNSCs-1, while NSC marker PAX6 was significantly downregulated. Moreover, after 2–3 weeks culture in neuron differentiation medium (NDM), astrocyte differentiation medium (ADM), or oligodendrocyte differentiation medium (ODM), hiNSCs-1 were able to differentiate into neurons (Tuj1, Map2, and NeuN-positive cells, Figure 3A–C), astrocytes (GFAP-positive cells, Figure 3D), and oligodendrocytes (Olig2, O4-positive cells, Figure 3E), respectively.

### 2.4. Differentiated Neurons from hiNSCs-1 Possess Mature Electrophysiological Properties as Bona Fide Neurons

**Figure 4:**
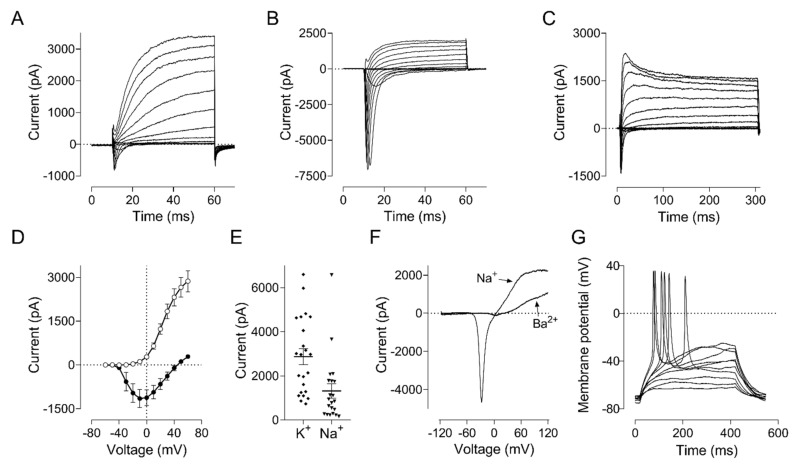
Electrophysiological properties of hiNSCs-1 cells. A, B, and C. Examples of membrane currents recorded in hiNSCs-1 cells in response to 50 ms (**A**,**B**) or 300 ms (**C**) voltage steps ranging from −60 mV to 60 mV, applied in 10 mV increments from a holding potential of −75 mV. (**D**) Average current-voltage plots of the Na+ (solid symbols) and K+ (clear symbols) currents constructed using recordings similar to those shown in panels (**A**–**C**) (*n* = 22). (**E**) Scatter plots of the absolute peak amplitudes of Na+ and K+ currents shown in panel (**D**). (**F**) Membrane currents recorded in response to 100 ms voltage ramps ranging from −120 to 120 mV before and after replacement of 145 mM NaCl in the bath solution with 100 mM BaCl2. (**G**) Action potentials recorded in a typical hiNSCs-1 cell in response to depolarizing current injections in the current clamp mode G. Action potentials recorded in a typical hiNSCs-1 cell in response to depolarizing current injections in the current clamp mode. Data were from 4 cell preparations.

To investigate if hiNSCs-1 can differentiate into functional neurons which have electrophysiological properties of mature neurons, hiNSCs-1 were subjected to whole-cell patch clamping after treating with MMC and being cultured in NDM for 5–6 weeks. The electrophysiology analysis in voltage clamp mode showed that hiNSCs-1 cells exhibit voltage-dependent membrane currents typical of neuronal cells (Figure 4A–C). The peak amplitude of the inward Na+ current ranged between −250 pA and −7000 pA (average −1150 ± 307 pA, *n* = 22) and the maximum amplitude of the outward K+ current at 60 mV ranged between 750 pA and 6500 pA (average 2900 ± 360 pA, *n* = 22) (Figure 4D,E). Application of a longer voltage protocol (300 ms) revealed the presence of at least two types of voltage gated K+ channels in these cells, responsible for the delayed rectifier and inactivating (A-type) K+ currents (Figure 4C). Replacement of 145 mM NaCl in the bath solution with 100 mM BaCl2 confirmed that virtually all inward current is mediated by voltage gated Na+ channels with very small contribution by the Ca^2+^ channels (Figure 4F). In current clamp mode hiNSCs-1 fired bona fide action potentials in response to the injection of depolarizing current from a membrane potential of −75 mV (Figure 4G).

### 2.5. Generation of hiNSCs from Another Fibroblast Cell Line

**Figure 5:**
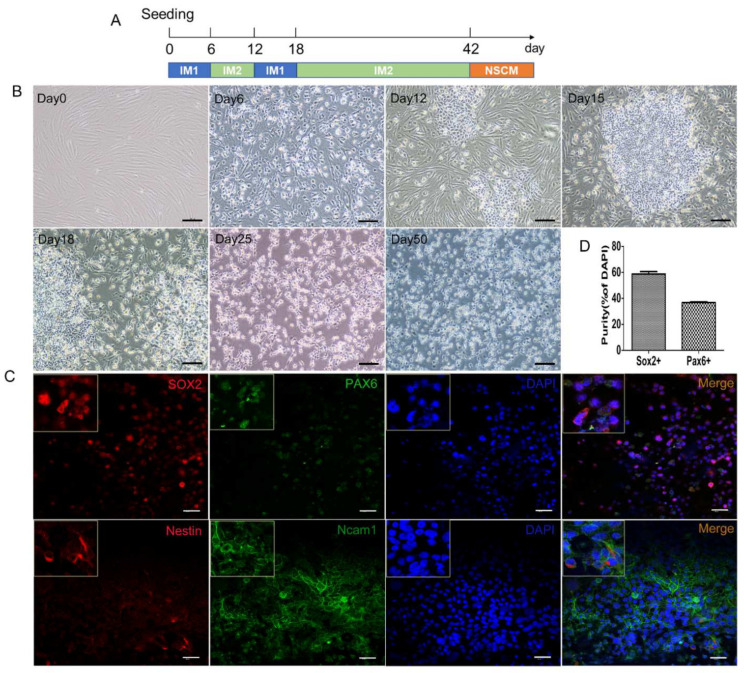

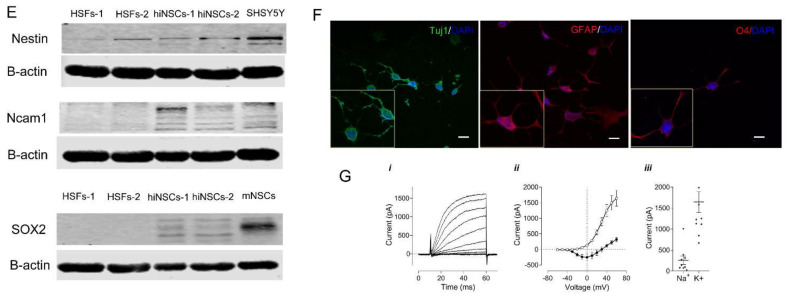
Generation of hiNSCs from another fibroblast cell line. (**A**) Scheme of induction procedure of hiNSCs-2. (**B**) Phase contrast images of cells during induction time, scale bars are 200 μm. (**C**) hiNSCs-1 stained for neural stem cell makers, SOX2, Nestin, PAX6, and Ncam1, scale bars are 40 μm. (**D**) The percentage of Sox2+ and Pax6+ cells in total cells at the time of quantification (means ± SEM, cell counting was from 3 triplicate samples) (**E**) Representative immunoblots for SOX2, Nestin (upper band), and Ncam1 in different cells, B-actin (beta-actin) was used as a loading control. (**F**) Differentiation of hiNSCs-2 in vitro, differentiated cells were immuno-positive for Tuj1, GFAP and O4 after 2–3 weeks culture in NDM, ADM, and ODM, respectively. (**G**) Electrophysiological properties of hiNSCs-2 derived neurons. *i*. Representative membrane currents recorded in hiNSCs-2 cell in response to the same voltage protocol as in Figure 4A. *ii*. Average current–voltage plots of the Na+ (solid symbols) and K+ (clear symbols) currents constructed using recordings similar to those shown in panels A (*n* = 9). *iii*. Scatter plots of the absolute peak amplitudes of Na+ and K+ currents shown in panel ii. Data were from 2 cell preparations.

To test if this induction protocol can generate similar results from other cell lines, we applied this protocol to another fibroblast cell line (HSFs-2) with a little difference in the induction procedure (Figure 5A). Similar to the results above, the induction protocol generated a distinct cell colony from HSFs in 12 days and these cells (termed hiNSCs-2) became morphologically stable and homogeneous after being cultured for months without small molecules (Figure 5B). Moreover, the immunostaining results showed that hiNSCs-2 were also immunoreactive for NSC markers Nestin, SOX2, PAX6, and Ncam1 (Figure 5C,D). The results from western blots also confirmed their upregulated expression of SOX2, Nestin and Ncam1 compared to HSFs-2 (Figure 5E). Furthermore, similar to hiNSCs-1, hiNSCs-2 could also differentiate into astrocytes, oligodendrocytes, and functional neurons when cultured in differentiation medium after being treated with MMC (Figure 5F,G). Together, these results have confirmed that our induction protocol can generate iNSCs from fibroblast cell lines.

## 3. Discussion

Recent advances in understanding of neural stem cells (NSCs) biology promise novel treatments of neurodegenerative diseases and other neurological disorders [18]. Although it is hard to obtain bona fide human NSCs, cell reprogramming has provided an opportunity to generate desired cells from other somatic cells. To counter the safety concerns of using viral transduction vectors or integrative transcription factors, here we have formulated a chemical cocktail of small molecules that can be used to convert human skin fibroblasts into iNSCs. These cells may be useful for neural disease modelling, drug screening, and even for treatment of neural diseases. To our knowledge, this is the first report of iNSCs generation from human skin fibroblasts with small molecules without any exogenous transcription factors.

Small molecules have been proven to be safe and effective for cell reprogramming including NSCs induction [17]. Although the mechanisms are not well understood, small molecules normally function as four roles which are epigenetic modifiers, signaling pathway regulators, metabolic modulators and nuclear receptor agonists or antagonists. Epigenetic modifiers and signaling pathway regulators are more widely used in reprogramming studies. In our protocol, Bix01294 (G9a histone methyltransferase inhibitor) and RG108 (DNA methyltransferase inhibitor) are epigenetic modifiers to reset the epigenetic memory of HSFs. CHIR99021 (Glycogen synthase kinase-3β inhibitor), Repsox (transforming growth factor-β inhibitor), LDN193189 (transforming growth factor-β inhibitor), and Y27632 (ROCK inhibitor) have been reported to be functional pathway regulators for NSCs induction [17] and are used to drive the “reset” cells to neural lineage. Ascorbic acid (antioxidant) and Q-VD-OPh (pan-caspase inhibitor) are used to reduce cell apoptosis. With this protocol, HSFs were converted into iNSCs which possessed similar morphology and gene expression as bona fide NSCs. Significantly, iNSCs were also able to differentiate into astrocytes, oligodendrocytes, and functional neurons. However, it is important to note that these iNSCs could remain proliferating for months without EGF and bFGF and that their differentiation was conditioned to the use of MMC in vitro. Although the fast proliferation rate facilitates the generation of sufficient cell numbers for research and possibly therapeutic, it may also bring some safety concerns. In addition, we have noticed that different cell lines reacted differently to the induction protocol (small molecules). In the above-mentioned two cell lines, although new colonies were appeared in both lines after 12 days, the second cell line had more dead cells than the first cell line at day 21. Due to the different functions of the two induction mediums, they should be changed according to the state of the cells but not strictly following a same protocol. In addition, we have tested this protocol in another cell line, but we have not got similar results as the cells appeared partially reprogrammed, which indicates the differences and hurdles between different cell lines for cell reprogramming. Therefore, further studies are required to understand better mechanisms regulating iNSCs induction, proliferation and differentiation, including their possible therapeutic properties in vivo conditions. 

Overall, the novel strategy described in this paper to generate iNSCs in vitro opens new possibilities for generating these cells in therapeutically suitable numbers which could facilitate their translation into the clinical settings.

## 4. Materials and Methods

### 4.1. Cell Culture

SHSY5Y cells were purchased from American Type Culture Collection (ATCC). Mouse neural stem cells (mNSCs) were retrieved from our storage. HSFs and SHSY5Y cells were expanded in HSF medium (Dulbecco’s modified Eagle’s medium [DMEM, Gibco, Scoresby, Australia], 10% fetal bovine serum [FBS] [Gibco, Scoresby, Australia], 1× Pen/Strep [Gibco, Scoresby, Australia]). mNSCs and hiNSCs were plated on culture dishes pre-coated with Geltrex (Gibco, Scoresby, Australia) and cultured in neural stem cell medium (NSCM) containing DMEM/F12 medium (Gibco) with 1× Pen/Strep (Gibco, Scoresby, Australia), 1× B27 supplement, 20 ng/mL recombinant human bFGF (R&D Systems, Melbourne, Australia), and 20 ng/mL recombinant human EGF (R&D Systems, Melbourne, Australia) for expansion.

### 4.2. Generation of iNSCs from Human Fibroblasts

On day 0, fibroblasts were seeded on a T-25 flask (1.2–1.5 × 10^6^ cells/flask) and cultured with Induction Medium 1 (IM1). Then, culture medium was changed with new prepared IM1 on day 3. On day 6, cells were transferred onto a T-25 flask pre-coated with Geltrex (Gibco, Scoresby, Australia) and cultured with Induction Medium 2 (IM2). Culture medium was changed with new prepared IM2 on day 9, then switched to IM1 on day 12. After several “IM1-IM2” cycles, iNSCs were cultured in NSCM for expansion. Stepwise induction protocol for hiNSCs-1 is provided in Supplemental Experimental Procedures. Detailed information about all culture medium is provided in Table S1. Information about all reagents used in this study is listed in Table S2.

### 4.3. In Vitro Differentiation of iNSCs

For spontaneous differentiation, iNSCs were seeded onto a T25 flask (2 × 10^6^ cells/flask) pre-coated with Geltrex and cultured in NSCM. On the next day, mitomycin C (MMC, 10 ug/mL) was added to NSCM for 1 h. Then, cells were cultured in NSCM without EGF and bFGF (termed as spontaneous differentiation medium, SDM). After 10 days, total RNA was extracted from cells for RT-PCR assay. For neuron, astrocyte, and oligodendrocyte differentiation, iNSCs were seeded onto 24-well plates (5 × 10^5^ cells/ well) pre-coated with Geltrex (for astrocyte differentiation) or laminin (for neuron and oligodendrocyte differentiation) and cultured in NSCM. On the next day, MMC (10 ug/mL) was added to the cells for 1 h. Then, culture medium was switched to neuron differentiation medium (NDM), astrocyte differentiation medium (ADM), and oligodendrocyte differentiation medium (ODM), respectively, until immunocytochemistry staining. NDM was half-changed every 2 days, and ADM and ODM were fully-changed every 2 days. Detailed information about all culture medium is provided in Table S1.

### 4.4. Immunocytochemistry Staining

Immunocytochemistry staining procedures were adjusted from an online protocol (https://www.abcam.com/protocols/immunocytochemistry-immunofluorescence-protocol, accessed on 20 December 2019). Briefly, cells on coverslips were fixed with 4% paraformaldehyde for 15 min and washed three times with ice-cold 1× phosphate buffered saline (PBS). Cells were then permeabilized with PBS containing 0.3% Triton X-100 for 15 min and washed in PBS three times, 5 min each wash (permeabilization is for intracellular proteins only). Cells were then incubated with 1% BSA, 22.52 mg/mL glycine in PBST (PBS+ 0.1% Tween 20) for 30 min to block unspecific binding of the antibodies. Then, cells were incubated in the diluted primary antibody in 1% BSA in PBST overnight at 4 °C. On the next day, cells were washed three times in PBS, 5 min each wash and then incubated with the secondary antibody and 0.1–1 μg/mL DAPI (DNA stain) in 1% BSA for 1 h at room temperature in the dark. After incubation, cells were washed again with PBS, three times for 5 min each. Cells were then mounted and viewed under a fluorescent microscope. All the detection antibodies applied here are listed in Table S3.

### 4.5. Western Blot

Cells were lysed with RIPA buffer (50 mM Tris-HCl (pH 7.4), 150 mM NaCl, 0.1%SDS, 2 mM EDTA, 1% Na-deoxycholate and 1% NP-40) containing cocktail inhibitors (protease and phosphatase) (1:500, Roche, Sydney, Australia). Then, samples were cleared by centrifugation at 14,000 rpm for 20 min. Total protein concentration was quantified using BCA assay (Thermo Fisher Scientific, Scoresby, Australia). Proteins were then denatured at 96 °C for 6 min after adding 5× SDS-PAGE protein loading buffer (MyBioSource, San Diego, CA, USA). A total of 15 µg of proteins of each sample were separated by gel electrophoresis on 10–14% SDS-polyacrylamide gels for 90 min at 110 volts using CBS gel system (C.B.S Scientific, San Diego, CA, USA), then the proteins were transferred onto a 0.45 µm nitrocellulose membrane (GE Healthcare, Parramatta, Australia) at 0.6 amps for 90 min. The blots were air-dried for 1 h before blocking with 5% bovine serum albumin (BSA)/tris buffered saline-tween (TBST) +0.05% azide (Sigma, Sydney, Australia). After blocking, the membranes were incubated with respective primary antibodies overnight at 4 °C. On the next day, the blots were washed with TBST (three times, 5 min each wash) and then incubated with corresponding secondary antibodies for 1 h at room temperature then washed again with TBST (three times, 5 min each wash). Later, the blots were visualized using Odyssey CLX imaging system (LI-COR Biosciences, Lincoln, NE, USA). As our Nestin antibody (Cat# MAB5326, Millipore, Sydney, Australia) does not react with mouse, we have selected SHSY5Y (a human neuroblastoma cell line which express Nestin and Ncam1) as a positive control for Nestin and Ncam1. However, As SHSY5Y barely express Sox2, we have selected mNSCs as a positive control for Sox2. All the detection antibodies applied here are listed in Table S3.

### 4.6. Real-Time qPCR

Real-time qPCR was carried out as previously described [19]. Total RNA was extracted from cells using RNeasy Mini Kit (Qiagen, Clayton, Australia), DNA contamination was further removed using turbo DNAse (Invitrogen, Scoresby, Australia). RNA was then transcribed into cDNA using cDNA Synthesis Kit (Bio-Rad, Galesville, Australia). qPCR was performed using iTaq Universal SYBR Green Supermix (Bio-Rad, Galesville, Australia) or iTaq Universal Probes Supermix (Bio-Rad, Galesville, Australia) on CFX Connect Real-Time System (Bio-Rad, Galesville, Australia). Samples were loaded in triplicate. GAPDH was used as a house-keeping gene. Final data were analyzed using the 2^−ΔΔCt^ method and were presented as relative fold changes versus fibroblasts or iNSCs (when fibroblasts were negative) as control. The primers and probes are shown in Table S4.

### 4.7. RNA-Sequencing

Total RNA was extracted from fibroblasts and iNSCs using RNeasy Mini Kit (Qiagen). Then RNA samples were sent to Shanghai Biozeron Biotechnology Co., Ltd., Shanghai, China for RNA-sequencing and analysis. The positive control, human neural progenitor cells (hNPCs) were taken from datasets from NCBI (GEO accession: GSM2073121). The datasets for HSFs-1 and hiNSCs-1 have been uploaded to Sequence Read Archive (SRA), NCBI. HSFs-1: SRR17024208; hiNSCs-1: SRR17024207.

### 4.8. Whole-Cell Patch Clamping

Whole-cell patch clamping was performed at room temperature using a computer-based patch-clamp amplifier (EPC-9, HEKA Electronics, Reutlingen, Germany) and PULSE software (HEKA Electronics). The bath solution contained (mM): NaCl 140; KCl 4; CaCl_2_ 2; MgCl_2_ 2; glucose 10; and HEPES, 10; adjusted to pH 7.4 with NaOH. The internal solution contained (mM): K gluconate 80; KCl 40; CaCl_2_ 2; EGTA 5; MgATP 2; and HEPES 10; adjusted to pH 7.3 with KOH. Patch pipettes were pulled from borosilicate glass and fire-polished. When filled with the internal solution, pipette resistance ranged between 2 and 4 MΩ. Series resistance did not exceed 10 MΩ and was compensated by 50–70%. Acquired currents were filtered at 2.7 kHz and sampled at 10 kHz. All voltages shown are nominal and have not been corrected for the liquid junction potential. The holding potential was −75 mV throughout. Cell capacitance was compensated automatically by the EPC-9 amplifier.

### 4.9. Statistical Analysis

All quantified data were statistically analyzed and presented as means ± SEM.

One-way ANOVA and Tukey’s Multiple Comparison Test were used to calculate statistical significance with *p* values.

## Data Availability

Data from this study is secured in a password protected computer. The RNA-sequencing datasets for HSFs-1 and hiNSCs-1 have been uploaded to Sequence Read Archive (SRA), NCBI. HSFs-1: SRR17024208; hiNSCs-1: SRR17024207.

## Supplementary Materials

The following supporting information can be downloaded at: https://www.mdpi.com/article/10.3390/ijms23031740/s1.

## References

[1] McGinley L.M., Kashlan O.N., Bruno E.S., Chen K.S., Hayes J.M., Kashlan S.R., Raykin J., Johe K., Murphy G.G., Feldman E.L. (2018). Human neural stem cell transplantation improves cognition in a murine model of alzheimer’s disease. Sci. Rep..

[2] Yasuhara T., Kameda M., Sasaki T., Tajiri N., Date I. (2017). Cell therapy for parkinson’s disease. Cell Transpl..

[3] Kokaia Z., Darsalia V. (2018). Human neural stem cells for ischemic stroke treatment. Results Probl. Cell Differ..

[4] Takahashi K., Yamanaka S. (2006). Induction of pluripotent stem cells from mouse embryonic and adult fibroblast cultures by defined factors. Cell.

[5] Li W., Wei W., Zhu S., Zhu J., Shi Y., Lin T., Hao E., Hayek A., Deng H., Ding S. (2009). Generation of rat and human induced pluripotent stem cells by combining genetic reprogramming and chemical inhibitors. Cell Stem Cell.

[6] Bellin M., Marchetto M.C., Gage F.H., Mummery C.L. (2012). Induced pluripotent stem cells: The new patient?. Nat. Rev. Mol. Cell Biol..

[7] Rao M.S., Malik N. (2012). Assessing ipsc reprogramming methods for their suitability in translational medicine. J. Cell Biochem..

[8] Shahbazi E., Mirakhori F., Ezzatizadeh V., Baharvand H. (2018). Reprogramming of somatic cells to induced neural stem cells. Methods.

[9] Han D.W., Tapia N., Hermann A., Hemmer K., Hoing S., Arauzo-Bravo M.J., Zaehres H., Wu G.M., Frank S., Moritz S. (2012). Direct reprogramming of fibroblasts into neural stem cells by defined factors. Cell Stem Cell.

[10] Xiao D.C., Liu X.N., Zhang M., Zou M., Deng Q.Q., Sun D.Y., Bian X.T., Cai Y.L., Guo Y.A., Liu S.T. (2018). Direct reprogramming of fibroblasts into neural stem cells by single non-neural progenitor transcription factor ptf1a. Nat. Commun..

[11] Miura T., Sugawara T., Fukuda A., Tamoto R., Kawasaki T., Umezawa A., Akutsu H. (2015). Generation of primitive neural stem cells from human fibroblasts using a defined set of factors. Biol. Open.

[12] Mirakhori F., Zeynali B., Rassouli H., Salekdeh G.H., Baharvand H. (2015). Direct conversion of human fibroblasts into dopaminergic neural progenitor-like cells using tat-mediated protein transduction of recombinant factors. Biochem. Biophys. Res. Commun..

[13] Yang H., Zhang L.L., An J., Zhang Q., Liu C.C., He B.R., Hao D.J. (2017). Microrna-mediated reprogramming of somatic cells into neural stem cells or neurons. Mol. Neurobiol..

[14] Zheng J., Choi K.A., Bang P.J., Hyeon S., Kwon S., Moon J.H., Hwang I., Kim Y.I., Kim Y.S., Yoon B.S. (2016). A combination of small molecules directly reprograms mouse fibroblasts into neural stem cells. Biochem. Biophys. Res. Commun..

[15] Cheng L., Hu W.X., Qiu B.L., Zhao J., Yu Y.C., Guan W.Q., Wang M., Yang W.Z., Pei G. (2014). Generation of neural progenitor cells by chemical cocktails and hypoxia. Cell Res..

[16] Han Y.C., Lim Y., Duffieldl M.D., Li H., Liu J., Manaph N.P.A., Yang M., Keating D.J., Zhou X.F. (2016). Direct reprogramming of mouse fibroblasts to neural stem cells by small molecules. Stem Cells Int..

[17] Liu D., Pavathuparambil Abdul Manaph N., Al-Hawwas M., Zhou X.F., Liao H. (2018). Small molecules for neural stem cell induction. Stem Cells Dev..

[18] Liu D., Bobrovskaya L., Zhou X.F. (2021). Cell therapy for neurological disorders: The perspective of promising cells. Biology.

[19] Liu D., Rychkov G., Al-Hawwas M., Manaph N.P.A., Zhou F., Bobrovskaya L., Liao H., Zhou X.F. (2020). Conversion of human urine-derived cells into neuron-like cells by small molecules. Mol. Biol. Rep..

