# Supplementary material for: Conversion of Human Fibroblasts into Induced Neural Stem Cells by Small Molecules
Source: Int J Mol Sci. 2022 Feb 3;23(3):1740. doi: 10.3390/ijms23031740 (PMC8835839; doi:10.3390/ijms23031740)
Supplement: Supplementary file 1 [file ijms-23-01740-s001.zip › ijms-1547719-supplementary.pdf]

Table S1. Components of all culture medium used in this study.

Culture medium	Components
HSF medium	DMEM supplemented with 10% fetal bovine serum and 1 x Pen/Strep
Neural stem cell medium (NSCM)	DMEM/F12 supplemented with 1 x Pen/Strep, 1 x B27, 20ng/ml bFGF and 20ng/ml EGF.
Induction medium 1 (IM1)	DMEM supplemented with 10% FBS, 1x Pen/Strep, 1x GlutaMAX, 1x MEM-NEAA, 10 μM RG108, 1 μM Bix01294, 3 μM CHIR99021, 1 μM Repsox, 0.2mM Ascorbic acid, 0.5μM LDN193189, 10μM Y-27632, 20ng/ml bFGF and 5 μM Q-VD-OPh
Induction medium 2 (IM2)	DMEM supplemented with 10% FBS, 1x Pen/Strep, 1x GlutaMAX, 1x MEM-NEAA, 10μM Y-27632, 20ng/ml bFGF and 20ng/ml EGF (5 μM Q-VD-OPh was added to IM2 when replating the cells)
Spontaneous differentiation medium (SDM)	DMEM/F12 supplemented with 1 x Pen/Strep, 1 x B27
Neuron differentiation medium-conditioned medium (NDM-CM)	Neurobasal Medium supplemented with 1 x Pen/Strep, 1 x B27, 1x GlutaMAX, and 20ng/ml BDNF. The medium was added to mNSCs for 24-48 hours, medium was then collected and centrifuged to remove cell debris.
Neuron differentiation medium-Brainphy (NDM-B)	BrainPhys™ Neuronal Medium supplemented with 1 x SM1 Neuronal Supplement, 1 x N2 Supplement-A, 100 μM cAMP-Na, 20ng/ml BDNF, 0.2mM Ascorbic acid and 10 μM Q-VD-OPh
Neuron differentiation medium (NDM)	NDM-CM: NDM-B= 1: 1
Astrocyte differentiation medium (ADM)	DMEM supplemented with 3% fetal bovine serum, 1 x Pen/Strep, 1x GlutaMAX, 1 x N2 and 10 μM Q-VD-OPh
Oligodendrocyte differentiation medium (ODM)	Neurobasal Medium supplemented with 1 x Pen/Strep, 1 x B27, 1x GlutaMAX, 30 ng/mL T3 and 10 μM Q-VD-OPh

Table S2. Basic medium and reagents used in this study.

Basic medium or reagent	Company	Cat #
DMEM	Life Technologies	11965084
DMEM/F12	Life Technologies	11320033
Neurobasal	Life Technologies	2110304
BrainPhys™ Neuronal Medium	StemCell Technologies	05793
SM1 Neuronal Supplement	StemCell Technologies	05711
N2 Supplement-A	StemCell Technologies	07152
penicillin/streptomycin	Life Technologies	15140122

Fetal Bovine Serum (FBS)	Life Technologies	10100139
B27	Life Technologies	17504044
N2	Life Technologies	A1370701
GlutaMAX	Life Technologies	35050061
MEM NEAA	Life Technologies	11140050
Bix01294	Reagents Direct	74-O89
RG108	Reagents Direct	41-L99
CHIR99021	Sigma	SML 1046
Ascorbic acid	Sigma	A5960
Repsox	Sigma	R0158
LDN193189	cayman chemicals	11802
Y27632	Sigma	Y0503
Q-VD-OPh	MedChemExpress	HY-12305
cAMP-Na	Sigma	A6885
EGF	R&D Systems	236-EG
bFGF	R&D Systems	233-FB
Geltrex	Life Technologies	A1413202
Laminin	Life Technologies	23017015
BDNF	Made in our lab	

Table S3. Detection antibodies.

Antibodies	Company	Cat #	Dilution (Immunocytochemistry)	Dilution (Western Blot)
Mouse anti SOX2	Biolegend	656102	1:100	1:500
Mouse anti Nestin	Millipore	MAB5326	1:100	1:400
Rabbit anti Pax6	Abcam	ab195045	1:100	
Goat anti Ncam1	R&D Systems	AF2408-SP	1:25	1:250
Chicken anti-Tuj1	Merck Millipore	AB9354	1:1000	
Rabbit anti-MAP2	Osenses	OSC212-1	1:1000	
Rabbit anti-NeuN	Abcam	ab177487	1:300	
Rabbit anti-GFAP	Dako	Z0334	1:200	
Mouse anti-O4	Sigma	MAB345	1:25	
Rabbit anti Olig2	Millipore	ab9610	1:500	
Mouse anti-B-actin	Abcam	ab8227		1:10000

Donkey anti-chicken CY2	Jackson Immunoresearch	JI703225155	1:1000	
Donkey anti-rabbit CY3	Jackson Immunoresearch	JI711165152	1:1000	
Donkey anti-rabbit CY2	Jackson Immunoresearch	JI711225152	1:1000	
Donkey anti-sheep CY2	Jackson Immunoresearch	JI713225147	1:1000	
Donkey anti-mouse CY3	Jackson Immunoresearch	JI715165150	1:1000	
Donkey anti-mouse CY2	Jackson Immunoresearch	JI715225150	1:1000	
Goat anti-mouse IgG	Li-Cor Biosciences			1:20000

Table S4. Primers and probes.

Gene	Forward	Reverse	Probe
Sox2	TACAGCATGATGCAGGACCA	GTACTGCAGGGCGCTCAC	CAGATGCAGCCCATGCAC
PAX6	GAGACTGGCTCCATCAGACC	TTTCCCAAGCAAAGATGGAC	AACCGAGAGTAGCGACTCCA
Nestin	GTCCATCCTCAGTGGGTCAG	AGCAGCTCATTCTGCTCCTC	GGGAGGAGTCGTTTCAGATG
Olig2	AGCTGCGACGACTATCTTCC	CGCTGCTGCCCTTACTCC	GAGCCCGATGACCTTTTTCT
L1cam	CGTGTCACCTACCAGAACCA	TCCACGGTGACATAGTACGC	GAGGAGGATGATGGCGAGTA
MAPT	GTCCGTACTCCACCCAAGTC	ATTCTTCAGGTCTGGCATGG	AGAGCCGCCTGCAGACAG
Map2	CCAATGGATTCCCATACAGG	TCTCCGTTGATCCCATTCTC	AGGATGAAGAGGGTGCCTTT
CNTN1	GATGAAACCATGAGCCCTTC	GGCTGTAAGGTCCATCTCCTT	AAGTCAAGGCCTTCAACAACA
NeuN	CACCCCACGCAGGACTAC	TGCTGGTGTGTACAGGGTCA	GGTCCCCACAGAGCATGG
Col1a1	TACCATGACCGAGACGTGTG	AGATCACGTCATCGCACAAC	GATCTGCGTCTGCGACAAC
GAPDH	ACCCAGAAGACTGTGGATGG	GAGGCAGGGATGATGTTCTG	GGGAAACTGTGGCGTGAT
NeuroD1	GGTGCCTTGCTATTCTAAGACGC	GCAAAGCGTCTGAACGAAGGAG	
GFAP	CCTGCAGATTCGAGAAACCAG	TCCTGCCTCACATCACATCC	
Tuj1	AGATCGGGGCCAAGTTCTG	TCGAGGCACGTACTTGTGAG	
S100A4	CTCTCCTCAGCGCTTCTTCTT	ACAGCAGTCAGGATCAACAC	
GAPDH	GTCTCCTCTGACTTCAACAGCG	ACCACCCTGTTGCTGTAGCCAA	

Supplemental Experimental Procedures

Generation of hiNSCs-1 from human fibroblasts

Day 0: Initial fibroblasts were seeded onto T-25 flasks (1.2-1.5 x 10^6 cells/flask) and cultured with IM1.

Day 3: Culture medium was changed with new prepared IM1.

Day 6: T-25 flasks were coated with 0.5-1% Geltrex (Gibco) for at least 2 hours. Cells were then transferred onto the T-25 flask and cultured with IM2 (with 5 µM Q-VD-OPh).

Medium change and cell transfer after Day 6 are shown as following:

Day 9: IM2. Day 12: IM1. Day 15: IM1. Day 18: IM2. Day 21: IM1. Day 25: IM1. Day 27: IM2.

Day 30: Cells were transferred onto T-25 flasks pre-coated with Geltrex (Gibco) and cultured IM2 (with 5 µM Q-VD-OPh).

Day 33: IM2. Day 37: IM2. Day 41: Cells were transferred onto T-25 flasks pre-coated with Geltrex (Gibco) and cultured IM2.

Day 42: Cells were then cultured in NSCM for expansion.
